# Divergent *Cryptosporidium* species and host-adapted *Cryptosporidium canis* subtypes in farmed minks, raccoon dogs and foxes in Shandong, China

**DOI:** 10.3389/fcimb.2022.980917

**Published:** 2022-08-22

**Authors:** Weijian Wang, Yanting Wei, Shuhui Cao, Wenjie Wu, Wentao Zhao, Yaqiong Guo, Lihua Xiao, Yaoyu Feng, Na Li

**Affiliations:** Guangdong Laboratory for Lingnan Modern Agriculture, Center for Emerging and Zoonotic Diseases, College of Veterinary Medicine, South China Agricultural University, Guangzhou, China

**Keywords:** *Cryptosporidium*, mink, raccoon dog, fox, subtype, zoonotic

## Abstract

*Cryptosporidium* spp. are common parasitic pathogens causing diarrhea in humans and various animals. Fur animals are widely farmed in Shandong Province, China, but the prevalence and genetic identity of *Cryptosporidium* spp. in them are unclear. In this study, 1,211 fecal samples were collected from 602 minks, 310 raccoon dogs and 299 foxes on two farms in Shandong and analyzed for *Cryptosporidium* spp. by nested PCR and sequence analyses of the small subunit rRNA gene. The overall infection rate of *Cryptosporidium* spp. was 31.5% (381/1,211), with a higher infection rate in raccoon dogs (37.7%, 117/310) than in foxes (32.4%, 97/299) and minks (27.7%, 167/602). By age, the highest infection rates of *Cryptosporidium* spp. were observed in raccoon dogs of 1-2 months, minks of 5-6 months, and foxes of > 12 months. Three *Cryptosporidium* species and genotypes were detected, including *C. canis* (*n* = 279), *C. meleagridis* (*n* = 65) and *Cryptosporidium* mink genotype (*n* = 37). Among the three major host species, raccoon dogs were infected with *C. canis* only (*n* = 117), while foxes were infected with both *C. canis* (*n* = 32) and *C. meleagridis* (*n* = 65), and minks with *C. canis* (*n* = 130) and *Cryptosporidium* mink genotype (*n* = 37). Subtyping of *C. canis* by sequence analysis of the 60 kDa glycoprotein gene identified eight subtypes. They belonged to two known subtype families, XXa and XXd, and two novel subtype families XXf and XXg, with host adaptation at the subtype family level. Notably, *C. canis* from foxes was genetically distant from those in other hosts. Further subtyping analysis identified three subtypes (IIIeA21G2R1, IIIeA19G2R1 and IIIeA17G2R1) of *C. meleagridis* and two novel subtype families Xf and Xg of the *Cryptosporidium* mink genotype. The presence of zoonotic *C. canis* subtypes in raccoon dogs and *C. meleagridis* subtypes in foxes suggests that these fur animals might be potential reservoirs for human-pathogenic *Cryptosporidium* spp.

## Introduction

*Cryptosporidium* spp. are common parasitic pathogens causing moderate-to-severe diarrhea in humans and various domestic and wild animals (Checkley et al., 2015). They are responsible for significant morbidity and mortality in immunocompromised people, young children, and neonatal animals (Collaborators, G. B. D. D. D, 2017; Santin, 2020). Humans can be infected with *Cryptosporidium* spp. by the fecal-oral route *via* contaminated food and water and direct contact with infected persons or animals (Yang et al., 2021). *Cryptosporidium hominis*, *C. parvum*, *C. meleagridis*, *C. canis* and *C. felis* are the most common human-pathogenic species worldwide (Ryan et al., 2021).

Fur animals including minks, raccoon dogs and foxes are wild animals raised as new farm animals for their fur. China is the largest market for fur consumption and one of world’s leading suppliers of fur (Fenollar et al., 2021). In 2021, fur animal farming industry in China is worth $61 billion, therefore is a notable contributor to local growth and employment (Williams, 2021). Currently there are nearly 6 million employees in the fur industry of China. Overall, 95% of fur farms are in five provinces of Shandong (greatest concentration), Hebei, Liaoning, Heilongjiang, and Jilin (Gong et al., 2020). On fur animal farms, the prevalence of zoonotic pathogens could increase as a result of intensive animal farming and poor sanitary conditions, leading to concerns for both animal health and public health (Guo et al., 2021).

Altogether, 10 *Cryptosporidium* species (*C. canis*, *C. parvum*, *C. hominis*, *C. meleagridis*, *C. felis*, *C. ubiquitum*, *C. andersoni*, *C. suis*, *C. tyzzeri*, and *C. galli*) and four *Cryptosporidium* genotypes (mink genotype, muskrat genotype I, fox genotype, and ferret genotype) have been identified in minks, raccoon dogs and foxes worldwide (Barrera et al., 2020; Qian et al., 2020; Kvac et al., 2021). Among them, *C. canis*, *C. meleagridis*, *C. parvum* and mink genotype have been detected in farmed minks, raccoon dogs and foxes in China, with *C. canis* and mink genotype being the dominant ones (Zhang et al., 2016a; Zhang et al., 2016b; Yang et al., 2018; Qian et al., 2020). The *C. parvum* identified in minks belonged to the subtype IIdA15G1, while four subtype families of Xb-Xe were found in *Cryptosporidium* mink genotype (Zhang et al., 2016a; Yang et al., 2018; Qian et al., 2020). However, genetic diversity of *C. canis* and *C. meleagridis* in fur animals at the subtype level remains unclear.

In this study, we examined the occurrence and genetic identity of *Cryptosporidium* spp. in minks, raccoon dogs and foxes on two farms in Shandong Province, which has the most concentrated fur farming industry in China. The genetic diversity of *C. canis*, *C. meleagridis* and mink genotype in these animals and their zoonotic potential was also assessed.

## Materials and methods

### Sample collection

From June to October in 2019, a total of 1,211 fecal samples were collected from reared fur animals on two farms in Shandong Province, eastern China, including 602 from minks (*Neovison vison*) and 310 from raccoon dogs (*Nyctereutes procyonoides*) on farm 1, and 299 from foxes (*Vulpes lagopus*) on farm 2. All the animals sampled were kept in cages with some interactions between neighboring cages. Fur animals of < 6 months in age were kept in groups of 2-3 animals per cage, while animals of > 6 months were kept individually. Fresh fecal droppings from these animals were collected on the ground under each cage, with one fecal sample being collected from each cage. The animals were divided into three age groups: 1-2 months (*n* = 512); 5-6 months (*n* = 286); and > 12 months (*n* = 413). As newborn foxes were kept together with their mothers, samples from foxes of 1-2 months were not collected in this study. Fecal samples were stored in 2.5% potassium dichromate at 4°C before DNA extraction.

### DNA extraction

Prior to DNA extraction, approximately 300 mg fecal samples were washed twice with distilled water to remove potassium dichromate by centrifugation at 2,000 × *g* for 10 min. Genomic DNA was extracted from the sediment using the FastDNA Spin Kit for Soil (MP Biomedicals, Solon, OH, USA) as described (Jiang et al., 2005). The extracted DNA was stored at -20°C until being used in PCR analyses.

### Genotyping and subtyping of *Cryptosporidium* spp.

*Cryptosporidium* spp. were detected and genotyped by nested PCR and sequence analyses of the small subunit (SSU) rRNA gene (Xiao et al., 1999). The *C. canis*, *C. meleagridis* and *Cryptosporidium* mink genotype identified were further subtyped by nested PCR and sequence analyses of the 60 kDa glycoprotein (*gp60*) gene as previously described (Glaberman et al., 2001; Alves et al., 2003; Jiang et al., 2021). As all the *C. canis*-positive samples from foxes in this study could not be subtyped using the *gp60* primers previously designed (Jiang et al., 2021), we have redesigned nested PCR primers based on the *gp60* gene of *C. canis* in foxes. The primers used in primary and secondary PCR were Canis-Fox-gp60-F1 (5'-TTCACAGTCTACTTTGATGG-3') and Canis-Fox-gp60-R1 (5'-GTACTCGGAAGCGGTGTA-3'), and Canis-Fox-gp60-F2 (5'-GACCCGGACGTTACATTTGATGG-3') and Canis-Fox-gp60-R2 (5'-TCAGTTAGATATCACCCATTAA-3'), respectively. The PCR condition was the same as previously described with an annealing temperature of 52°C being used in both primary and secondary PCR (Jiang et al., 2021). Two replicates were used in PCR analysis of each target for each sample. Genomic DNA of *C. parvum* from cattle and reagent-grade water were used as the positive and negative control, respectively.

### Sequence analysis

All positive secondary PCR products of the SSU rRNA (~ 830 bp) and *gp60* (~ 850 bp) amplicons were sequenced bidirectionally using Sanger sequencing by Sangon Biotech (Shanghai, China). The nucleotide sequences obtained were assembled using ChromasPro 2.1.5 (http://technelysium.com.au/ChromasPro.html), edited using BioEdit 7.1.3 (http://www.mbio.ncsu.edu/BioEdit/bioedit.html), and aligned with each other and reference sequences downloaded from GenBank using ClustalX 2.0.11 (http://clustal.org). To assess the phylogenetic relationship among *Cryptosporidium* species/genotypes/subtypes, maximum likelihood trees were constructed using Mega 10.1.6 (http://www.megasoftware.net/). The general time-reversible model was used in substitution rate calculation and 1,000 replicates were used in bootstrapping analysis. The newly identified *Cryptosporidium* subtypes were named according to the established nomenclature system (Xiao and Feng, 2017; Jiang et al., 2021).

### Statistical analysis

Differences in the prevalence of *Cryptosporidium* spp. among age groups of fur animals were compared using the Chi-square test implemented in SPSS 20.0 (IBM Corp., Armonk, NY, USA). Differences were considered significant at *P* < 0.05.

## Results

### Occurrence of *Cryptosporidium* spp. in fur animals

All the fur animals sampled were clinically normal with no obvious signs of diarrhea. The overall prevalence of *Cryptosporidium* spp. in these animals was 31.5% (381/1,211). Raccoon dogs had the highest infection rate (37.7%, 117/310), followed by foxes (32.4%, 97/299) and minks (27.7%, 167/602). By age, minks of 5-6 months (55.8%) had significantly higher prevalence of *Cryptosporidium* spp. than minks of 1-2 months (22.6%; *χ* ^2^ = 38.281, *df* = 1, *P* = 0.000) and > 12 months (24.6%; *χ* ^2^ = 20.322, *df* = 1, *P* = 0.000). For raccoon dogs, *Cryptosporidium* infection rate in animals of 1-2 months (64.5%) was significantly higher than in those of 5-6 months (37.0%; *χ* ^2^ = 15.911, *df* = 1, *P* = 0.000) and > 12 months (9.0%; *χ* ^2^ = 22.134, *df* = 1, *P* = 0.000). In contrast, foxes of > 12 months (35.2%) had higher prevalence of *Cryptosporidium* spp. than foxes of 5-6 months (27.0%; *χ* ^2^ = 2.030, *df* = 1, *P* = 0.154) (**Table 1**).

**Table 1:** Occurrence of *Cryptosporidium* species/genotypes and subtypes in minks, raccoon dogs and foxes on two farms in Shandong, China.

Host	Age (month)	No. positive at*ssu* rRNA locus/No. sampled (%)	Species/genotype (*n*)	No. positive at *gp60* locus/No. sampled (%)	Subtype (*n*)		
					*C. canis*	*C. meleagridis*	Mink genotype
Mink	1-2	91/402 (22.6)	*C. canis* (82), mink genotype (9)	37/51 (72.5)	XXd2 (13), XXf1 (22), XXf2 (1)	–	XfA5G1R1(1)
	5-6	48/86 (55.8)	*C. canis* (23), mink genotype (25)	15/34 (44.1)	XXf1 (8)	–	XgA5G1R1 (7)
	> 12	28/114 (24.6)	*C. canis* (25), mink genotype (3)	8/12 (66.6)	XXd2 (1) XXf1 (7)	–	–
	Total	167/602 (27.7)	*C. canis* (130), mink genotype (37)	60/97 (61.9)	XXd2 (14), XXf1 (37), XXf2 (1)	–	XfA5G1R1(1), XgA5G1R1 (7)
Raccoon dog	1-2	71/110 (64.5)	*C. canis* (71)	33/35 (94.3)	XXa2 (1), XXa4 (32)	–	–
	5-6	37/100 (37.0)	*C. canis* (37)	14/14 (100)	XXa2 (8), XXa4 (6)	–	–
	> 12	9/100 (9.0)	*C. canis* (9)	6/6 (100)	XXa2 (4), XXa4 (2)	–	–
	Total	117/310 (37.7)	*C. canis* (117)	53/55 (96.4)	XXa2 (13), XXa4 (40)	–	–
Fox	5-6	27/100 (27.0)	*C. canis* (15), *C. meleagridis* (12)	15/27 (55.5)	XXg1 (3), XXg2 (5), XXg3 (4)	IIIeA21G2R1 (2), IIIeA17G2R1 (1)	–
	> 12	70/199 (35.2)	*C. canis* (17), *C. meleagridis* (53)	31/769 (44.9)	XXg1 (2), XXg2 (2), XXg3 (3)	IIIeA21G2R1 (18), IIIeA19G2R1 (6)	–
	Total	97/299 (32.4)	*C. canis* (32), *C. meleagridis* (65)	46/96(47.9)	XXg1 (5), XXg2 (7), XXg3 (7)	IIIeA17G2R1 (1), IIIeA19G2R1 (6), IIIeA21G2R1 (20)	–

### *Cryptosporidium* species/genotypes

All 381 *Cryptosporidium*-positive samples were successfully genotyped by sequence analysis of the SSU rRNA gene. Three *Cryptosporidium* species and genotypes were identified, including *C. canis* (*n* = 279), *C. meleagridis* (*n* = 65) and *Cryptosporidium* mink genotype (*n* = 37). Among them, *C. canis* was detected in all three host species, whereas *C. meleagridis* and mink genotype were detected alone in foxes and raccoon dogs, respectively. *C. canis* was the dominant species in minks (130/167, 77.8%) and raccoon dogs (117/117, 100%), while *C. meleagridis* was the dominant one in foxes (65/97, 67.0%) (**Table 1**).

**Figure 1:**
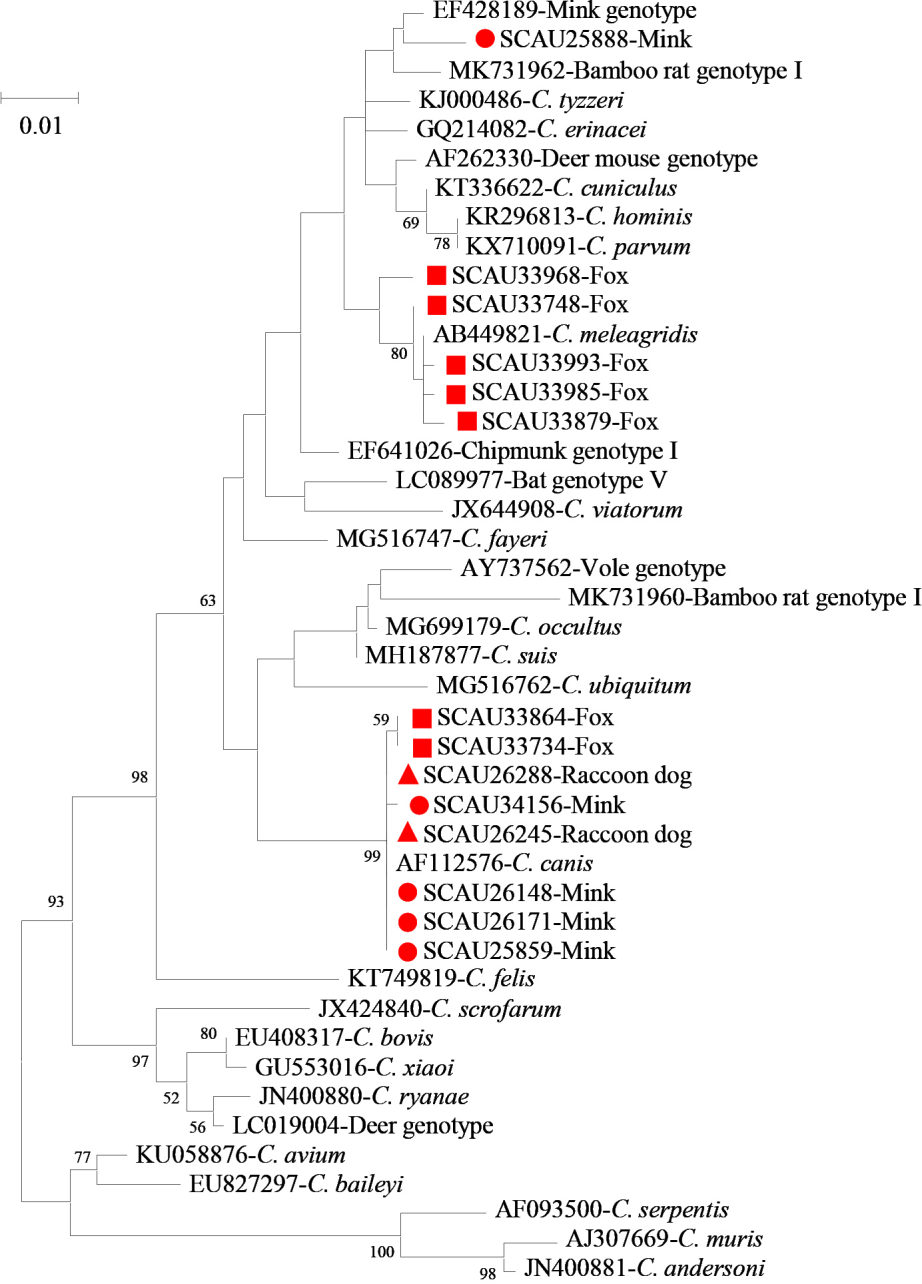
Phylogeny of *Cryptosporidium* spp. based on the maximum likelihood analysis of the partial SSU rRNA gene using the general time-reversible model for the calculations of substitution rates. Bootstrap values greater than 50% from 1,000 replicates are displayed. Samples detected in minks, raccoon dogs and foxes from this study are indicated with red circles, triangles, and squares, respectively.

Among the 279 *C. canis* samples, 232 from minks and raccoon dogs generated SSU rRNA sequences that were identical to the GenBank reference sequence AF112576. Nucleotide sequences of the remaining 15 *C. canis* samples from minks and raccoon dogs had minor differences from the reference sequence AF112576, including one single nucleotide polymorphism (SNP) in one sample and 3-18 nucleotide deletions in 14 samples. Likewise, nucleotide sequences of 32 *C. canis* samples from foxes had minor differences from the reference sequence AF112576, including 1 SNP and 4 nucleotide insertions in 28 samples and 1 SNP and 2 nucleotide insertions in four samples (**Figure 1**). Similarly, sequences of 65 *C. meleagridis* samples obtained from foxes had 0-2 SNPs compared with the reference sequence AB449821 (**Figure 1**). In contrast, all 37 sequences generated from *Cryptosporidium* mink genotype were identical to the reference sequence EF428189 from minks in China (**Figure 1**).

### Subtypes of *C. canis*

**Figure 2:**
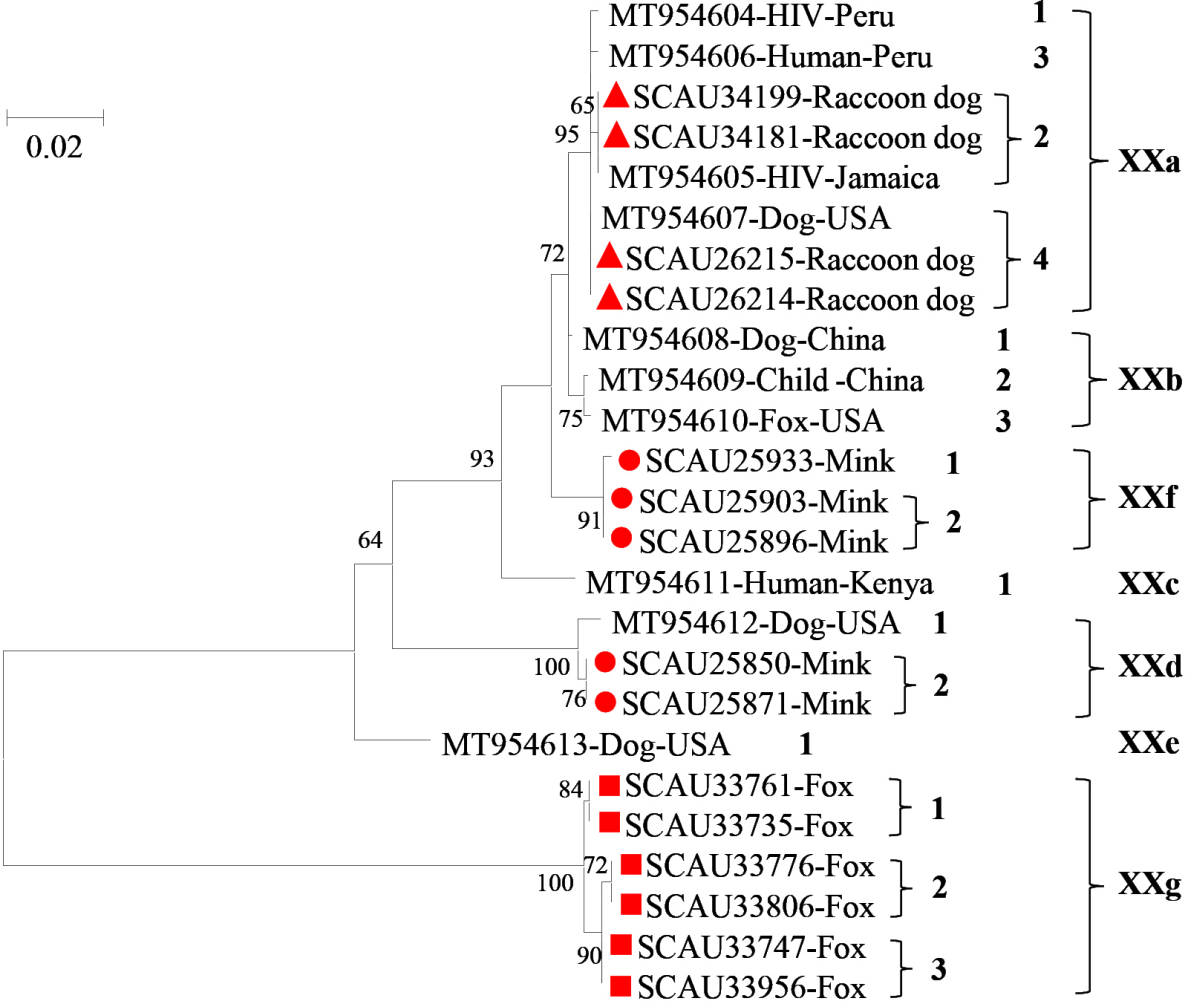
Phylogenetic relationship among seven *Cryptosporidium canis* subtype families (XXa-XXg) based on the maximum likelihood analysis of the partial *gp60* gene using the general time-reversible model for the calculations of substitution rates. Bootstrap values greater than 50% from 1,000 replicates are displayed. Samples detected in minks, raccoon dogs and foxes from this study are indicated with red circles, triangles, and squares, respectively.

A total of 148 *C. canis* samples were randomly selected from each age group of each host species for further subtyping at the *gp60* locus, with 124 samples (83.8%) being successfully amplified and sequenced. Among them, 52/61 samples (85.2%) from minks, 53/55 samples (96.4%) from raccoon dogs, and 19/32 (59.4%) samples from foxes were successfully subtyped. In these *C. canis* samples, two known subtype families of XXa and XXd and two new subtype families of XXf and XXg were identified. XXf had 92.6-98.5% nucleotide sequence identity to known subtype families XXa-XXe, while XXg had 66.8-69.8% nucleotide sequence identity to subtype families XXa-XXf. In phylogenetic analysis, XXf clustered with XXb, whereas XXg was placed outside the clade containing XXa-XXf (**Figure 2**).

Among the 52 *C. canis* samples from minks, two subtype families of XXd and XXf were identified, including three subtypes XXd2 (*n* = 14), XXf1 (*n* = 37) and XXf2 (*n* = 1) (**Table 1**). Nucleotide sequence of the new subtype XXd2 had 18 nucleotide insertions and 4 SNPs compared with the reference sequence MT954612 of subtype XXd1. In contrast, there was only 1 SNP between the nucleotide sequences of XXf1 and XXf2.

The 53 *C. canis* isolates from raccoon dogs all belonged to the XXa subtype family, including two known subtypes XXa2 (*n* = 13) and XXa4 (*n* = 40) (**Table 1**). The nucleotide sequences of XXa2 and XXa4 were identical to the reference sequences MT954605 and MT954607, respectively.

The 19 *C. canis* isolates from foxes all belonged to the new subtype family XXg, including three subtypes XXg1 (*n* = 5), XXg2 (*n* = 7) and XXg3 (*n* = 7) (**Table 1**). There were 1-3 SNPs and 4 nucleotide insertions/deletions among the nucleotide sequences of XXg1, XXg2 and XXg3.

### Subtypes of *C. meleagridis*

Of the 65 *C. meleagridis*-positive samples from foxes, 27 samples (41.5%) were successfully amplified and sequenced at the *gp60* locus. Only one known subtype family IIIe was identified, including three known subtypes IIIeA21G2R1 (*n* = 20), IIIeA19G2R1 (*n* = 6), and IIIeA17G2R1 (*n* = 1) (**Table 1**). The nucleotide sequences of IIIeA21G2R1, IIIeA19G2R1 and IIIeA17G2R1 were identical to the reference sequences KU852728, MG969387 and MG969388, respectively, with the exception of one isolate of IIIeA21G2R1 having 1 SNP compared with the reference sequence KU852728.

### Subtypes of *Cryptosporidium* mink genotype

**Figure 3:**
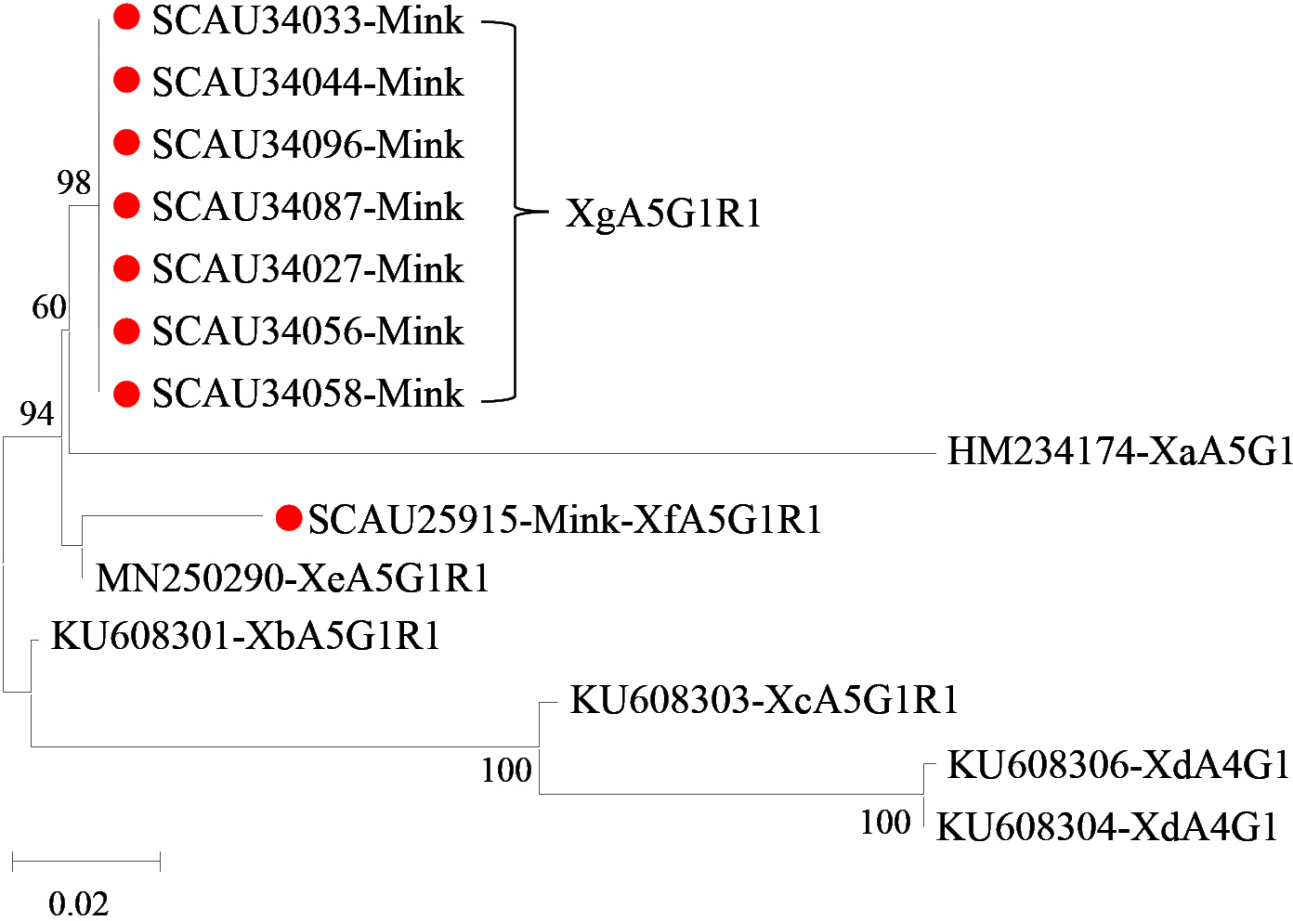
Phylogenetic relationship among seven subtype families (Xa-Xg) of *Cryptosporidium* mink genotype based on the maximum likelihood analysis of the partial *gp60* gene using the general time-reversible model for the calculations of substitution rates. Bootstrap values greater than 50% from 1,000 replicates are displayed. Samples detected in minks from this study are indicated with red circles.

Of the 37 samples of *Cryptosporidium* mink genotype, eight (21.6%) were successfully amplified and sequenced at the *gp60* locus. Among them, two new subtype families Xf and Xg were identified. Between them, Xf had 70.6-97.6% nucleotide sequence identity to known subtype families Xa-Xe, while Xg had 65.7-92.1% nucleotide sequence identity to subtype families Xa-Xf. In phylogenetic analysis, Xf clustered with Xe, whereas Xg was placed together with Xa in one clade but had the highest sequence identity to Xe (**Figure 3**). The Xf and Xg isolates belonged to subtypes XfA5G1R1(*n* = 1) and XgA5G1R1 (*n* = 7), respectively (**Table 1**).

## Discussion

Results of the present study indicate that *Cryptosporidium* spp. are common in fur animals on two farms in Shandong, China. The overall *Cryptosporidium* infection rate (31.5%) in this study was higher than those in most other studies worldwide (Klockiewicz et al., 2021). In China, it was also higher than previous reports of *Cryptosporidium* spp. in farmed fur animals sampled in Heilongjiang (7.4-22.9%), Jilin (7.2-23.1%), Liaoning (0.0%), Hebei (1.2-7.8%), and Xinjiang (12.2%) (Wang et al., 2008; Zhang et al., 2016a; Zhang et al., 2016b; Yang et al., 2018; Qian et al., 2020). By animal species, the *Cryptosporidium* infection rate in minks (27.7%) in this study was similar to that in Heilongjiang (29.6%), but was higher than those in other areas in China (1.2-12.1%) (Wang et al., 2008; Zhang et al., 2016a; Yang et al., 2018; Qian et al., 2020). Likewise, infection rates in raccoon dogs (37.7%) and foxes (32.4%) in this study were higher than those (0.0-20.5% and 1.6-15.9%, respectively) in previous reports in China (Zhang et al., 2016a; Zhang et al., 2016b; Yang et al., 2018; Qian et al., 2020). We have further noted differences in age-associated infection rates among farmed minks, raccoon dogs and foxes. The differences in infection rates among age groups in these animals are likely due to farming environment and animal management for different fur animals on the two study farms.

The dominant *Cryptosporidium* species and genotypes appears to be different among fur animals. In this study, *C. canis* was the dominant species in minks and raccoon dogs. This is similar to observations of most studies in China, except for two studies with mink genotype being dominant in minks (Wang et al., 2008; Qian et al., 2020). However, *C. meleagridis* was found to be dominant in foxes here, which is in contrast with previous reports showing that *C. canis* is more prevalent in farmed foxes (Zhang et al., 2016b; Yang et al., 2018). *C. canis* and *C. meleagridis* have been frequently found in humans in developing countries and are among the five most common human-pathogenic species of *Cryptosporidium* (Yang et al., 2021). Thus, practitioners in the fur industry should be educated on the zoonotic potential of the two *Cryptosporidium* species and their transmission routes.

By using the newly developed subtyping tool (Jiang et al., 2021), both high genetic diversity and host adaptation were observed within *C. canis* at the *gp60* locus in the present study. Four *C. canis* subtype families were identified in these animals. Among them, XXa was only detected in raccoon dogs, XXd and XXf only in minks, and XXg only in foxes, indicating the existence of host adaptation in *C. canis* among fur animal species. Similarly, apparent host-adapted *gp60* subtype families are present in some other human-pathogenic *Cryptosporidium* spp. such as *C. parvum*, *C. hominis* and *C. ubiquitum* (Feng et al., 2018). Nevertheless, the two subtypes XXa2 and XXa4 identified in raccoon dogs here have been reported in humans in the United States, Peru, Ethiopia, and Jamaica (Jiang et al., 2021). This indicates that *C. canis* in farmed raccoon dogs could have zoonotic potential.

*C. canis* from foxes appears to be genetically different from those in other hosts such as humans, dogs, minks and raccoon dogs. Our data indicate that the XXg subtype family identified in foxes is genetically distant from other subtype families in phylogenetic analysis of *gp60* sequences. Nucleotide sequences of the partial *gp60* gene obtained from XXg showed low identity to that of XXa-XXf, with substantial nucleotide differences in the 5′ region of the obtained sequence. This could be responsible for the failure of previously designed primers to amplify the *gp60* gene of *C. canis* from foxes (Jiang et al., 2021). In addition, the result supports the existence of genetic divergences among *C. canis* isolates from different hosts. The genetic differentiation at the subtype level might be the reflection of various transmission patterns and intensities of *C. canis*.

*C. meleagridis*, the dominant species found in foxes in this study, is a known zoonotic pathogen. The subtypes IIIeA19G2R1 and IIIeA17G2R1 identified in foxes here have been detected in chickens and birds in China (Liao et al., 2018; Liao et al., 2021; Lin et al., 2022) and humans in China, Uzbekistan and India (Stensvold et al., 2014; Lebbad et al., 2021). However, the dominant *C. meleagridis* subtype in foxes, IIIeA21G2R1, has been only reported previously in one patient in Indonesia (Lebbad et al., 2021). Therefore, despite the possible mechanic passages of *C. meleagridis* in foxes through food sources from chickens, farmed foxes could play a potential role in the transmission of this zoonotic pathogen.

In conclusion, results of this study indicate a common occurrence of *Cryptosporidium* spp. in minks, raccoon dogs and foxes on two farms in Shandong, China, and extend our knowledge on the genetic diversity and transmission of *Cryptosporidium* spp. in farmed exotic animals. Host adaptation was observed in *C. canis* from different fur animals. The presence of zoonotic *C. canis* subtypes in raccoon dogs and *C. meleagridis* subtypes in foxes suggests that these fur animals might be potential reservoirs for human-pathogenic *Cryptosporidium* spp. With the increased intensity in fur animal farming, attentions should be paid to the potential public health implications of contacts with fur animals and environmental contamination from fur animal farms.

## Data availability statement

The datasets presented in this study can be found in online repositories. The names of the repository/repositories and accession number(s) can be found below: Representative nucleotide sequences generated in this study were deposited in GenBank under accession numbers ON832696-ON832709 and ON863562-ON863575.

## Ethics statement

The animal study was reviewed and approved by Ethics Committee of the South China Agricultural University.

## Author contributions

YF and NL conceived and designed the experiments. WWa, YW, SC, WWu, and WZ performed the experiments and analyzed the data. YG and LX provided technical assistance. WWa, YW, YF, and NL wrote the manuscript. All authors contributed to the article and approved the submitted version.

## Funding

This work was supported by National Natural Science Foundation of China (U1901208 and 31972697), Laboratory of Lingnan Modern Agriculture Project (NT2021007), Natural Science Foundation of Guangdong Province (2021A1515011075), and Innovation Team Project of Guangdong University (2019KCXTD001).

## Conflict of interest

The authors declare that the research was conducted in the absence of any commercial or financial relationships that could be construed as a potential conflict of interest.

## Publisher’s note

All claims expressed in this article are solely those of the authors and do not necessarily represent those of their affiliated organizations, or those of the publisher, the editors and the reviewers. Any product that may be evaluated in this article, or claim that may be made by its manufacturer, is not guaranteed or endorsed by the publisher.
